# *Yersinia pestis* in *Pulex irritans* Fleas during Plague Outbreak, Madagascar

**DOI:** 10.3201/eid2008.130629

**Published:** 2014-08

**Authors:** Jocelyn Ratovonjato, Minoarisoa Rajerison, Soanandrasana Rahelinirina, Sébastien Boyer

**Affiliations:** Institut Pasteur de Madagascar, Antananarivo, Madagascar (J. Ratovonjato, S. Boyer);; World Health Organization Collaborating Centre, Institut Pasteur de Madagascar, Antananarivo (M. Rajerison, S. Rahelinirina)

**Keywords:** fleas, Pulex irritans, Yersinia pestis, plague, Madagascar, bacteria, outbreak, human flea, Enterobacteriaceae, rats

**To the Editor:** *Yersinia pestis* (family *Enterobacteriaceae*) is a bacterium that can cause high rates of death in susceptible mammals and can provoke septicemic, pneumonic, and bubonic plague in humans (*1*). This zoonotic pathogen can be transmitted directly by infectious droplets or by contact with contaminated fluid or tissue or indirectly through flea bites (*1*).

Plague was introduced into Madagascar in 1898 from rat-infested steamships that had sailed from affected areas (*2*). Now, Madagascar is 1 of 2 countries in Africa that have reported cases of human plague every year since 1991 (*3*). During January 2008–January 2013, the number of human plague cases reported in Madagascar ranged from 312 to 648 per year. Of these, 61.8%–75.5% were laboratory confirmed (National Plague Laboratory of the Ministry of Health, pers. comm.). Most (>83%) confirmed cases were bubonic plague, which most commonly results from flea bites, suggesting that these bites were the most common mode of *Y. pestis* transmission. In Madagascar, *Xenopsylla cheopis* fleas have been known as the primary plague vector in urban areas, whereas *Synopsyllus fonquerniei* fleas have been usually involved in plague transmission in rural areas (*2*). 

In January 2013, a total of 9 suspected bubonic plague cases, 3 confirmed, were reported in Soavina, a rural area in the district of Ambatofinandrana, Madagascar. Domestic fleas were collected with candle traps inside 5 houses during 3 nights (Table). Fleas were also caught on small mammals trapped inside houses and outside in the sisal fences and rice fields (Table). A total of 319 fleas belonging to 5 species in 5 genera were collected inside and outside the houses, an average of 44 per house (maximum 71): *Pulex irritans, Echidnophaga gallinacea*, and *Ctenocephalides canis* fleas were collected inside the houses (244, 76.5%), and *S. fonquerniei* and *X. cheopis* fleas were collected outside (75, 23.5%). The human flea, *P. irritans*, was the most collected flea species (233, 73.3%), followed by *S. fonquerniei* (62, 19.4%), *X. cheopis* (13, 4.1%), *E. gallinacea* (10, 3.1%), and *C. canis* (1, 0.3%).

**Table:** Fleas collected inside and outside houses in Soavina, Madagascar, January 2013

Species	Total no. (%)	No. (%) inside	No. (%) outside
*Pulex irritans*	233 (73.0)	233 (95.5)	0
*Ctenocephalides canis*	1 (0.3)	1 (0.4)	0
*Echidnophaga gallinacea*	10 (3.1)	10 (4.1)	0
*Synopsyllus fonquerniei*	62 (19.4)	0	62 (82.7)
*Xenopsylla cheopis*	13 (4.1)	0	13 (17.3)
Total	319 (100)	244 (100)	75 (100)

Bacterial DNA was extracted from 277 fleas of 5 species: 233 *P. irritans*, 24 *S. fonquerniei,* 9 *X. cheopis*, 10 *E. gallinacea*, and 1 *C. canis*. PCR to detect *Y. pestis* was performed by using primers YP1 (5′-ATC TTA CTT TCC GTG AGA AG-3′) and YP2 (5′-CTT GGA TGT TGA GCT TCC TA-3′) to amplify a 478-bp fragment (*4*). *Y. pestis* DNA was then amplified and genotyped by Beckman Coulter Genomics Inc. (Takeley, United Kingdom). The positive control was *Y. pestis* reference strain (strain 6/69, 3 × 10^8^ bacteria/mL; Institut Pasteur de Madagascar).

Detection of *Y. pestis* was carried out on 274 fleas belonging to 5 flea species: 230 *P. irritans* (181 unfed and 49 engorged), 24 *S. fonquerniei* (15 unfed and 9 engorged), 9 *X. cheopis* (8 unfed and 1 engorged), 10 *E. gallinacea* (blood-feeding status not identified), and 1 unfed *C. canis*. *Y. pestis* was detected in 9 *P. irritans* fleas (7 male [6 unfed and 1 engorged] and 2 [engorged] female) from 3 houses, including the house where a confirmed human case of plague had occurred (Technical Appendix). Eight sequences (GenBank accession nos. KJ361938–KJ361945) were obtained and share 99% nucleotide homology with plasminogen activator genes of *Y. pestis* published in GenBank (accession nos. AF528537, AY305870). No *Y. pestis* was detected in the 24 *S. fonquerniei,* 9 *X. cheopis,* 10 *E. gallinacea*, or 1 *C. canis* fleas collected. 

Although only *X. cheopis* and *S. fonqueniei* fleas had previously been described as plague vectors in Madagascar, *P. irritans* fleas were most commonly collected during this field study; engorged and unfed male and female *P. irritans* fleas carried *Y. pestis*. Other studies have found *P. irritans* fleas in the plague risk area in other countries in Africa (*5**,**6*); one study found that *P. irritans* fleas may play a role in plague epidemiology in Tanzania (*5*). Data on *P. irritans* fleas in rats make it unlikely that these fleas are involved in rat-to-human transmission of *Y. pestis* in Madagascar. During 1922–1995, a total of 118,608 rats were caught and examined in Madagascar, but only 148 *P. irritans* fleas were identified, and none have been found on rats since 1996 (http://www.pasteur.mg/spip.php?rubrique124). The high density of *P. irritans* fleas we observed in villages where plague outbreaks occurred in late 2012 and early 2013 (http://www.pasteur.mg/spip.php?rubrique124) supports the possibility that *P. irritans* fleas played a role in domestic human-to-human transmission of *Y. pestis* during these outbreaks.

Technical Appendix Agarose gel electrophoresis showing positive PCR results for *Yersinia pestis* in DNA of 9 *Pulex irritans* fleas collected from 3 houses in an area of Madagascar where plague outbreaks occurred in late 2012 and early 2013.
