# Supplementary material for: Yersinia pestis in Pulex irritans Fleas during Plague Outbreak, Madagascar
Source: Emerg Infect Dis. 2014 Aug;20(8):1414–5. doi: 10.3201/eid2008.130629 (PMC4111190; doi:10.3201/eid2008.130629)
Supplement: Technical Appendix — Agarose gel electrophoresis showing positive PCR results for Yersinia pestis in DNA of 9 Pulex irritans fleas collected from 3 houses in an area of Madagascar where plague outbreaks occurred in late 2012 and early 2013. [file 13-0629-Techapp-s1.pdf]

Article DOI: http://dx.doi.org/10.3201/eid2008.130629

Yersinia pestis in *Pulex irritans* Fleas during Plague Outbreak, Madagascar

Technical Appendix

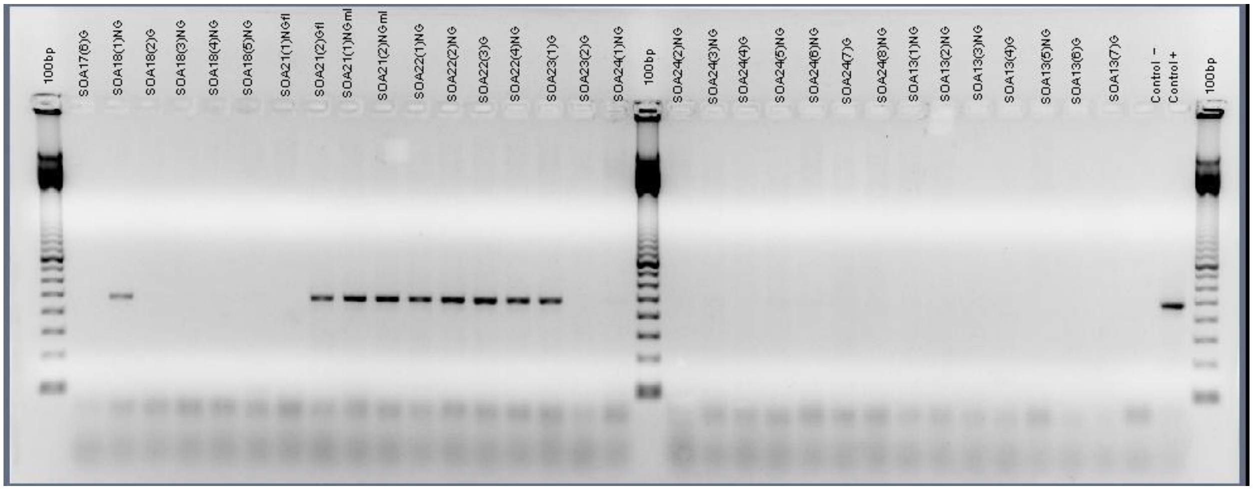

Technical Appendix Figure. Agarose gel electrophoresis showing positive PCR results for *Yersinia pestis* in DNA of 9 *Pulex irritans* fleas collected from 3 houses in an area of Madagascar where plague outbreaks occurred in late 2012 and early 2013. Expected size = 478 bp; 100 bp = base ladder.
